# Mayaro Virus Infection in Traveler Returning from Amazon Basin, Northern Peru

**DOI:** 10.3201/eid1804.111717

**Published:** 2012-04

**Authors:** Andreas Neumayr, Martin Gabriel, Jasmin Fritz, Stephan Günther, Christoph Hatz, Jonas Schmidt-Chanasit, Johannes Blum

**Affiliations:** Swiss Tropical and Public Health Institute, Basel, Switzerland (A. Neumayr, J. Fritz, C. Hatz, J. Blum);; Bernhard Nocht Institute for Tropical Medicine, Hamburg, Germany (M. Gabriel, S. Günther, J. Schmidt-Chanasit)

**Keywords:** Mayaro virus, Mayaro virus infection, traveler, viral polyarthritis, viral polyarthralgia, alphavirus infection, viruses, Amazon Basin, northern Peru

**To the Editor:** We report the case of a 27-year-old male Swiss tourist who spent 3.5 weeks (July 6–30, 2011) vacationing in the vicinity of Tarapoto, a small city located in the rainforests of the Amazon Basin in northern Peru. An acute febrile illness developed in the man during the second week of his stay. Signs and symptoms of illness were chills, malaise, frontal headache, generalized myalgia, a self-limiting painful cervical and inguinal lymphadenopathy (lasting ≈1 week), slowly progressive and pronounced polyarthralgic pains of the peripheral joints, and a transient nonpruritic maculopapular rash (starting at the forearms ≈1 week after onset of fever and spreading to the trunk and later to the neck and face before fading after 3 days).

The traveler sought care at the local hospital, where physicians diagnosed suspected dengue fever on the basis of the clinical signs and symptoms, and received symptomatic treatment with paracetamol. The fever and other signs subsided within 1 week, except the arthralgia, which did not improve. The polyarthritis initially was accompanied by swelling of the affected joints and showed a symmetric pattern, mainly affecting the small joints of the hands and feet as well as the wrists, ankles, and knees.

After returning home to Switzerland, the patient consulted his general practitioner (August 1, 2011) because of persisting, incapacitating joint pains. The patient reported stiffness of the affected joints, mainly in the morning and after immobility. Physical examination of the affected joints did not reveal visible clinical signs of inflammation (swelling, redness, effusion). Laboratory tests were performed for complete blood count, liver and kidney function, C-reactive protein, and serologic testing for dengue virus, chikungunya virus, *Borrelia burgdorferi*, *Chlamydia trachomatis*, Epstein-Barr virus, parvovirus B19, *Salmonella* Typhi, and *S.* Paratyphi, but none revealed a cause for the symptoms. Over almost 2 more months, the joint pains did not improve; thus, the patient was referred to a rheumatologic clinic and subsequently to the Swiss Tropical and Public Health Institute, Basel, Switzerland, for evaluation of a putative travel-related cause of the polyarthralgia.

Because of the patient’s travel history, the course of the illness and clinical signs and symptoms experienced during the journey, and the evolution and characteristics of the persisting joint pains, we suspected an underlying Mayaro virus (MAYV) infection. Serologic testing (indirect immunofluorescence and virus neutralization assays) for several alphaviruses were performed as described (*1*), and the results (Table) confirmed our presumptive diagnosis.

**Table:** Results of serologic testing for a Mayaro virus–infected Swiss traveler returning from the Amazon Basin, northern Peru, 2011*

Virus	Antigenic complex	Indirect immunofluorescence assay						Virus neutralization assay	
		First serum sample†			Second serum sample‡			First serum sample†	Second serum sample‡
		IgG	IgM		IgG	IgM			
Mayaro virus	SF	2,560	1,280		2,560	40		40	160
Chikungunya virus	SF	160	<20		160	<20		ND	ND
O`Nyong-nyong virus	SF	20	<20		80	<20		ND	ND
Ross River virus	SF	160	<20		160	<20		ND	ND
Semliki Forest virus	SF	80	<20		80	<20		20	20
Barmah Forest virus	BF	20	<20		20	<20		ND	ND
Venezuelan equine encephalitis virus	VEE	80	<20		80	<20		ND	ND
Western equine encephalitis virus	WEE	80	<20		80	<20		ND	ND
Sindbis virus	WEE	160	<20		160	<20		<20	<20
Eastern equine encephalitis virus	EEE	<20	<20		<20	<20		ND	ND

*SF, Semliki Forest; ND, not done; BF, Barmah Forest; VEE, Venezuelan equine encephalitis; WEE, Western equine encephalitis; EEE, Eastern equine encephalitis. †Sample obtained August 29, 2011. ‡Sample obtained September 12, 2011.

Several viral infections (e.g., dengue, rubella, parvovirus B19, hepatitis B, hepatitis C, HIV, and human T-lymphotropic virus type 1) can be accompanied by arthralgia. However, the most prominent and long-lasting polyarthritic symptoms occur in patients infected by alphaviruses (family *Togaviridae*).

Alphaviruses are arthropod-borne viruses (arboviruses) that circulate among a wide variety of wild animals in relative mosquito vector–specific and host-specific enzootic cycles; infection in humans (dead-end hosts) is almost exclusively incidental. Clinical cases and virus isolation have been reported only from northern South America, where MAYV circulates in an enzootic sylvatic cycle (similar to that for yellow fever) involving forest-dwelling *Haemagogus* spp. mosquitoes as vectors and nonhuman primates as natural hosts (*2*). Infections in humans mostly occur sporadically, are strongly associated with occupational or recreational exposure in rainforest environments, and are assumed to represent spillover from the enzootic cycle (*2*).

MAYV was first isolated in Trinidad in 1954; since then, sporadic cases, clusters, outbreaks, and small epidemics of Mayaro fever have been reported from Brazil, Bolivia, Columbia, French Guiana, Guyana, Peru, Venezuela, and Surinam (*3*). In addition to the clinical cases and virus isolates reported from northern South America, serologic survey findings suggest the presence of MAYV in Costa Rica, Guatemala, and Panama (*4*) (Figure A1).

Clinical signs of this acute, dengue-like, febrile illness last 3–7 days and typically include chills, headache, retro-orbital and epigastric pain, myalgia, arthralgia, nausea, vomiting, diarrhea, and a maculopapular rash (sometimes followed by desquamation) (*7*). However, as with other alphavirus infections (i.e., chikungunya [Africa, Asia], o’nyong-nyong [Africa], Ross River [Australia, Oceania], Barmah Forest [Australia], and Sindbis [Africa, Europe, Asia, Australia]), the hallmark of MAYV infection is the highly debilitating arthralgia. Permanent damage of the affected joints has not been reported. Hemorrhagic manifestations of MAYV infections are rare but have been described (*3*). Concerns over the potential emergence of urban transmission of MAYV were raised after a laboratory study showed vector competence of *Aedes aegypti* mosquitoes (*8*).

International travelers are rarely given a diagnosis of MAYV infection (*9*,*10*). This might be attributed to the overall low frequency of the infection; the dengue-like signs and symptoms, which may lead to a misdiagnosis; and the fact that the disease is not well known outside MAYV-endemic regions. Physicians treating patients with signs and symptoms of a dengue-like illness and a recent history of travel to MAYV-endemic areas should consider MAYV infection in the differential diagnosis, especially if arthralgia is prominent and prolonged and dominates the clinical picture.

## Figures and Tables

**Figure A1:**
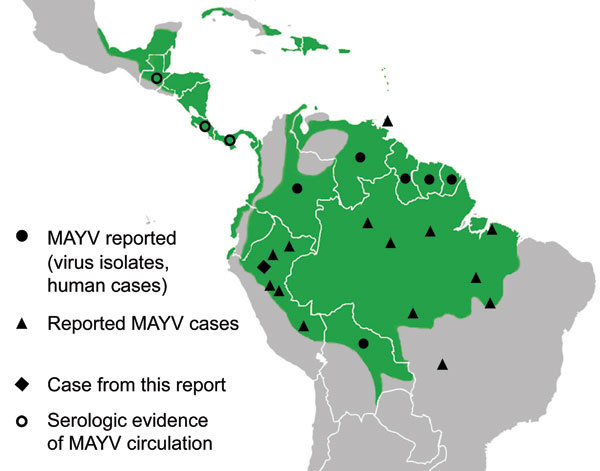
Distribution of Mayaro virus (MAYV) in the Central and South America (*3**,**5**–**7*).
